# Nanomicellar Extraction of Polyphenols—Methodology and Applications Review

**DOI:** 10.3390/ijms222111392

**Published:** 2021-10-21

**Authors:** Paweł Śliwa, Karolina Śliwa

**Affiliations:** Faculty of Chemical Engineering and Technology, Cracow University of Technology, Warszawska St. 24, 31-155 Kraków, Poland

**Keywords:** micellar-mediated extraction, cloud point extraction, non-ionic surfactants, biosurfactants, IL-type surfactants, polyphenols

## Abstract

The selection of the appropriate extraction method is crucial, especially for the receiving of active substances from plant material. The extraction using supercritical liquids and micellar-mediated extraction (MME) is the most advantageous among the alternative methods to classical solid–liquid extraction. However, the latter seems to be the best solution when the desired actives are polar. The following article presents a comprehensive review of the micellar-mediated extraction method in the last decade. The theoretical principle of the process was also refreshed and the current state of knowledge on the applications for analytical and manufacturing purposes was summarized.

## 1. Introduction

Various methods of liquid–liquid, liquid–solid, supercritical, ultrasound, and microwave-assisted extraction methods have been used in the industry as well as in the laboratory for many years. Some of them, however, have several disadvantages: they are expensive, require the use of large amounts of toxic and flammable solvents, give low efficiency, or require the use of expensive equipment or a large expenditure of electricity and heat [1]. Moreover, plant extracts are raw materials for cosmetic or pharmaceutical products, and the organic solvents used for herb infusions, e.g., ethanol or ethyl acetate, additionally have a strong drying effect on the skin. Therefore, in order to eliminate the potentially irritating and sensitizing effects of plant extracts, and solvents as well, methods should be carefully selected.

An alternative to the classical methods is the extraction using supercritical liquids, such as supercritical CO_2_. The method enables the yield of non-polar compounds at a low process temperature of above 34 °C (critical parameters: T_c_ = 304.2 K (30 °C), p_c_ = 7.38 MPa) [2]. While polar compounds could be eluted with supercritical water, the disadvantage of this method is the high process temperature of about 374 °C (critical parameters: T_c_ = 647.3 K (373.1 °C), p_c_ = 22.0 MPa), which may lead to a dissociation of compounds or loss of thermolabile substances. The micelle-mediated extraction (MME) and also its improvement using the ultrasonic (UAMME) or microwave (MAMME) field are also unconventional methods of obtaining actives from plant material [3,4]. In the MME, instead of harmful organic solvents, an aqueous surfactant solution is used, with which it is possible to solubilize the desired components [3,5,6]. Besides the lack of toxic organic eluents, such as methanol, ethyl acetate, and propanol, the great advantages of that methodology is the short extraction time and low cost of the process [6]. Additionally, the application of various micellar systems allows the increasing of the selectivity of obtaining the preferred group or groups of chemical compounds, and in that way the elimination of potentially allergenic substances [7]. It is a completely safe method, non-toxic for humans as well as environmentally friendly, and therefore belongs to the group of methods satisfying the principles of “Green Chemistry”. The MME suits the cosmetic industry perfectly because the applied surfactants are commonly used as co-emulsifier in the emulsions, and so in that manner, MME could be regarded as waste-less.

In the MME methods, non-ionic surfactants are most often used because of their excellent solubilizing properties and the lack of toxicity. Generally, these compounds are classified as harmless reagents and acceptable for use in cosmetic preparations [6]. Contrary to ionic surfactants, non-ionic ones have a low critical micelle concentration (CMC) so they can be used in low concentrations [3]. The micellar-mediated extraction is proceeded at low, usually room temperature. Most non-ionic surfactants, when heated above a temperature known as the turbidity point, decrease their solubility in water and the formation of two phases is observed. This phenomenon is used in the cloud point extraction (CPE). This method has high efficiency of isolated natural substances, higher than in the conventional method, and therefore is more often used as a sample extraction/preconcentration in analytical chemistry. For the first time, such a methodology was used for the enrichment of analytes in environmental studies, such as for the determination of polycyclic aromatic hydrocarbons in seawater and to determine trace amounts of heavy metals or toxins in biological samples [8]. However, further studies showed that MME can also be successfully used to extract biologically active substances such as vitamins, paracetamol, flurbiprofen, salicylic acid [3], or polyphenols [3,7,9,10,11]. Moreover, it was proved that compounds solubilized by the micelle were protected against oxidation [1]. Due to its excellent efficiency, environmental friendliness, low cost, and waste-less properties, the ultrasonic micelle-mediated extraction method appeared to be one of the best technologies available for obtaining flavonoid-rich extracts [11].

**Figure 1:**
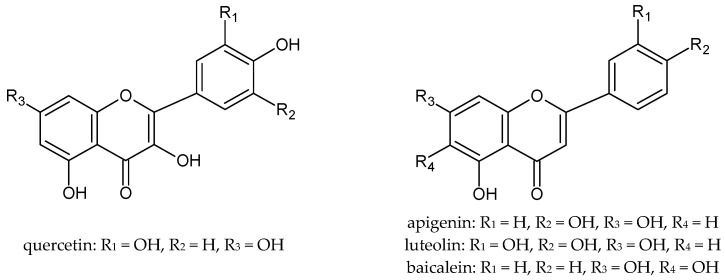
The chemical structure of selected flavonoids.

The antioxidant properties of plant extracts determine their potential regenerative and anti-inflammatory effects on the skin. Therefore, these raw materials are successfully used in cosmetic and medicinal preparations for the care of various types of skin. It is well known that plant extracts owe their properties mainly to the polyphenols they contain. Polyphenols, even at low concentrations, protect against oxidation and delay this process to a large extent. The antioxidant activity of polyphenols is due to their low redox potential, which allows them to act as reducing agents, by hydrogen- or electron-donating and thus scavenging free radicals [12]. Among the polyphenols, flavonoids are of particular importance (Figure 1), which are characterized by a diversity of structure and multidirectional biological activity as well as excellent antioxidant properties. Flavonoids scavenge free radicals by either the single electron transfer mechanism (SET) or the hydrogen atom transfer mechanism (HAT) [13]. The route of radical reactions depends on the composition of the extract and the chemical structure of the antioxidants [14]. Glycosides in vitro may show little activity, but it has been proven that in biological systems these compounds can undergo enzymatic hydrolysis, which results in the formation of an active aglycone [15].

The following review summarizes the research on the use of surfactants in the extraction of polyphenols. The issue was divided into two main topics, resulting from the applications of MME, i.e., extraction as a step of separation and/or preconcentration of analytes and the use of micellar-assisted extraction to obtain new raw material for cosmetics, pharmaceuticals, or food. The review, preceded by a theoretical background, deals with some methodological improvements and new applications as well as giving advantages and limitations of the MME. Summarization of the computational findings are also provided. The referenced literature covers mainly the years 2010–2020.

## 2. Theoretical Background

### 2.1. Micellization and Aggregation of Surfactants

**Figure 2:**
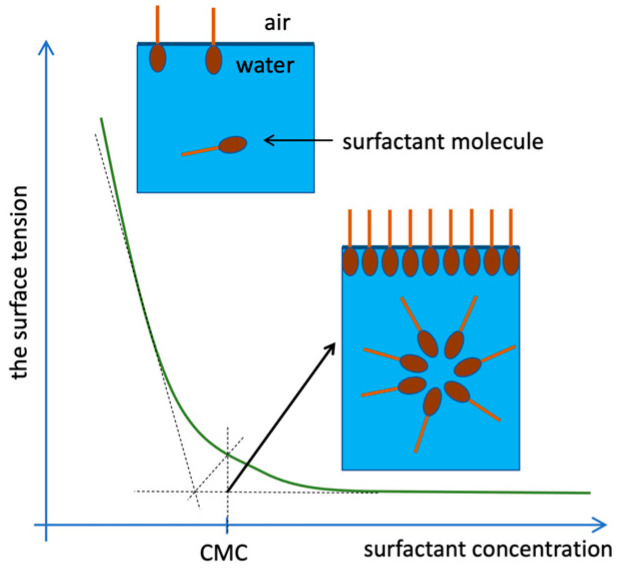
Arrangement of surfactant molecules in the solution depending on the concentration [17].

In dilute aqueous solutions, surfactants occur mainly in the form of monomers, and less often dimers or trimers, i.e., in the form of individual molecules freely suspended in the volume of the solution. A proportional decrease in the surface tension of the solution is observed as the concentration of the surfactant increases until the concentration reaches a specific limit, referred to as the critical micellization concentration (CMC, Figure 2). At this point, the surfactant molecules self-assemble to form larger aggregates [16].

The process of micellization of surfactants in an aqueous solution is a spontaneous phenomenon, because it is associated with the reduction of the free energy of the system. The driving force of the process is the desire of surfactants to limit the contact surface of the hydrophobic part of molecules with water [18]. The micellization process in water can be considered as the balance of non-covalent intermolecular forces, such as electrostatic interactions, hydrogen bonds, and Van der Waals interactions (hydrophobic, spatial) [19].

**Figure 3:**
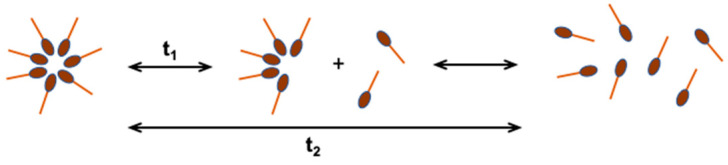
Dynamic equilibrium of micelles with monomers in aqueous solution; t_1_—fast relaxation time, in the order of microseconds, t_2_—slow relaxation time, in the order of milliseconds [20].

Micelles are ordered aggregates containing from a dozen to even 100 molecules of surfactant, which in an aqueous solution are in dynamic equilibrium with monomers (Figure 3). The concentration of non-aggregated particles in the water phase is close to the CMC value [16,20,21]. Depending on the geometrical structure of the surfactant, their aggregates can be spherical, cylindrical, double-layered, or inverted. The shape and size of micelles can be controlled by changing the chemical structure of the surfactant or the conditions of solution preparation (temperature, pH, surfactant concentration, ionic strength) [22].

### 2.2. Effect of the Surfactant Structure and Environmental Conditions on the Micellization

The micellization process is influenced by factors such as the type and concentration of the surfactant, the type of solvent, the process temperature, pressure, pH, the presence of electrolytes, and the presence of other organic substances [23]. After exceeding the CMC value, some physical properties of the solution begin to change rapidly, such as surface tension (γ), osmotic pressure (π), molar conductivity (κ) in the case of ionic surfactants, solubilization capacity, and turbidity [23,24,25].

In the case of non-ionic surfactants, the CMC decreases with the increasing length of the alkyl chain, as well as the number of non-polar groups in the structure of its hydrophilic part [26]. As the temperature increases, the CMC value of the ionic surfactants in the aqueous solution initially decreases until it reaches a minimum, and then begins to increase again. For surfactants such as alkyl mono ethers of ethoxylated glycols, this minimum is in the temperature range of 40–50 °C. At the same time, the corresponding minimum CMC value increases as the degree of ethoxylation of the molecule increases [27]. The addition of salt reduces the critical micellar concentration of ionic and, to a lesser extent, non-ionic surfactants. It is known that the addition of salt to the solution of non-ionic surfactants increases the degree of aggregation and the size of the micelles, and consequently reduces the CMC [24]. The CMC value can be modified by adding also organic compounds to the solution [28]. For non-ionic ethoxylated surfactants, the CMC value depends on the pH of the solution and increases with increasing pH at a constant temperature [12].

### 2.3. Solubilization

Solubilization is the spontaneous formation of a stable, isotropic solution of a substance that is insoluble or slightly soluble in water in an aqueous micellar solution [20]. The solubilization process is accomplished through the formation of so-called microemulsions, as aqueous surfactant solutions form macroscopically homogeneous but microscopically heterogeneous systems. Hydrophobic substances are solubilized in the non-polar micelle core, while polar compounds can form mixed micelles by partially integrating into the structure of micellar aggregates [29,30].

Solubilization is of great importance, for example, in pharmacy because many active substances are insoluble or very poorly soluble in water. Moreover, the process reduces the degree of degradation of these substances, as well as increases their bioavailability and minimizes their side effects. The solubility of the solute is low when the surfactant concentration is less than its critical micellar concentration. However, above the CMC, the solubility increases linearly with increasing surfactant concentration [22].

### 2.4. Effect of the Solubilized Compound, Surfactant Structure, and Environmental Conditions on the Solubilization

The research by Morisue et al. [31] shows that the solubilization of aromatic hydrocarbons is controlled primarily by lipophilic interactions and the location of the hydrophobic active substance in the core of surfactant micelles depends on the compound hydrophobicity [31]. Liu et al. [32] also confirm that the location of the active substance in the micelle is controlled by its hydrophobicity. Moreover, it has been proven that the solubilization efficiency increases with the increase of surfactant concentration [32]. The opposite effect is observed in reverse micelle systems, which are formed in non-polar solvents. The formation of a stable micelle in such a medium is determined by the presence of water near lipophilic groups. In reverse micelles, primarily proteins are solubilized [33]. Compounds with a high affinity to water may be located in the middle of the micellar aggregate; however, only when they are in dissociated form [34].

The efficiency of solubilization depends also on the length of the hydrocarbon chain of the surfactant molecule. With the increasing number of carbon atoms in the surfactant, the hydrophobicity of the medium increases, which improves the solubility of the active substance. This property has been confirmed for both ionic and non-ionic surfactants [35]. However, usually considering the type of surfactant, non-ionic surfactants appear to be better solubilizing agents as they most often have a lower critical micellar concentration, are less toxic, and are very effective at low concentrations [20]. It has been proven, however, that the use of a mixture of non-ionic and ionic surfactants increases the extraction efficiency of polar organic compounds [36]. When hydrocarbons or weakly polar molecules with a long hydrophobic chain are solubilized, the yield tends to increase with the increasing length of the hydrophobic surfactant chain. Increasing the hydrophobic chain length in the solubilized molecules in most cases reduces the efficiency of the solubilization process. It has also been proven that unsaturated compounds are more easily solubilized than their saturated analogues [20]. Numerous studies also show that the solubilization of aromatic compounds is much greater than that of aliphatic compounds [22].

In the case of temperature dependence, generally, increases cause an increase of the solubilization efficiency, because it leads to greater solubility in water of actives, as well as to an increase of micelle size [20]. In the case of non-ionic ethoxylates surfactants, the opposite effect is observed, which could be related to the increased dehydration of oxyethylene groups and reduction of available spaces between hydrophobic chains [22]. Additionally, a too high temperature can lead to thermal decomposition of the extracted substance [26].

The addition of salt usually increases the solubilization efficiency of the ionic surfactant solution, as it leads to an increase of micelle size and a decrease of the CMC [20]. The pH of the solution is also a factor influencing the solubilization of the substance, as it changes the equilibrium between the ionized and molecular forms of the active substance. The highest solubilization efficiency could be achieved at the pH value for which the solubilized substance remains in a non-ionized form [22].

### 2.5. Micellar-Mediated Extraction at the Cloud Point

**Figure 4:**
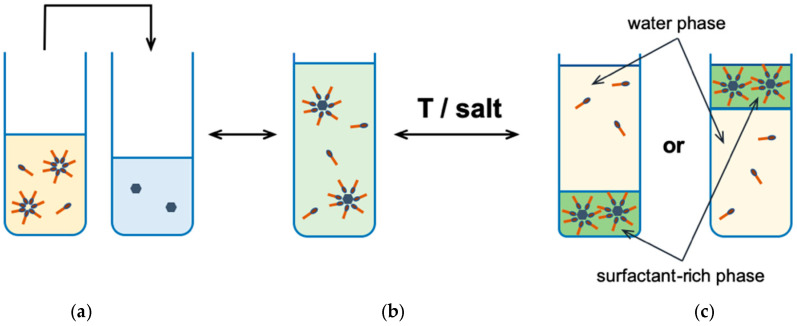
The cloud point extraction: (**a**) an analyte solution; (**b**) an analyte solubilized in micelles after adding surfactant solution; (**c**) a phase separation after temperature change or by adding salt [40].

Micellar extraction at the cloud point (CPE, cloud point extraction, Figure 4), is based on the phase separation phenomenon at or above some characteristic temperature for non-ionic surfactant called the cloud point (CP). At this point, its aqueous solution becomes cloudy [8,37], due to light scattering in the visible range [29]. The cloud point is a characteristic value for a given surfactant and is within a fairly wide temperature range depending on the type of surfactant [38]. Phase separation by heating the system above the cloud point can be applied to almost all non-ionic surfactants, but also some mixtures of anionic and cationic surfactants [16,39,40]. The organic phase (coacervate) is formed by a surfactant with an extracted compound and is a so-called surfactant or surfactant-rich phase. The aqueous phase, in this case, is the precipitate in which the concentration of the surfactant is close to the CMC value.

In the case of non-ionic surfactants, the coacervate is obtained by heating the solution above the cloud point, while in the case of zwitterionic compounds, by lowering the temperature. For example, in the case of polyoxyethylene surfactants as a result of increasing the temperature, the hydrogen bonds between the oxygen atoms of the oxyethylene units and water molecules are broken [41]. In many applications, there is a need to separate the surfactants from the coacervate, which is performed by dialysis or hydrophobic adsorption [42]. It has been shown that the phase separation procedure is reversible and after restoring the initial conditions, the micelles can again form a homogeneous system [1]. Due to the different densities of individual layers, centrifugation is used to accelerate their separation. In case the surfactant phase has a higher density than the aqueous phase, it is preferable to cool the sample, which increases the viscosity of the micelles, and the surfactant phase is better attached to the bottom of the test tube. Subsequently, the aqueous phase is decanted and the residual solvent is evaporated [26,43].

### 2.6. Effect of the Process Parameters on the CPE

The CPE efficacy depends basically on the same factors as was pointed out for MME (the concentration of the given tenside solution, salt addition, pH, the presence of polymers or other organic compounds) [16,29]. The cloud point decreases with an increasing hydrophobicity, i.e., with the increasing number of carbon atoms in the alkyl chain or a decrease of the oxyethylene units number [39,41]. The cloud point of the surfactant solution is additive, which means that for a mixture of different surfactants, it is the weighted average of the CP of all ingredients [41,44]. The addition of small amounts of cationic or anionic surfactants to the non-ionic surfactant solution causes a significant increase in the CP value [45]. Another important factor in CPE extraction is the pH of the solution. The best extraction efficiency of dissociable substances is obtained at a pH for which the predominant form of the analyte is neutral [43,46]. The effect of electrolyte addition on the cloud point of non-ionic surfactant is also known. The presence of Na^+^ and K^+^ cations lowered the cloud point, causing dehydration of the oxyethylene units [41]. Anions have the main influence on salinity, while cations affect non-ionic compounds to a lesser extent. Anions can be arranged according to the strength of the salting-out into the so-called Hofmeister series: SO_4_^2−^ > HPO_4_^2−^ > F^−^ > Cl^−^ > Br^−^ > NO_3_^−^ > I^−^ > ClO_4_^−^ > SCN^−^ [47]. For most non-ionic surfactants, the presence of salt facilitates phase separation as it increases the ionic strength of the water and changes its density [44].

### 2.7. Examples of the CPE Applications

Micellar-mediated extraction is a clean, safe, ecological method. The isolated active substances can be safely used as ingredients in cosmetics, drugs, and food products. Moreover, the methodology of this process is easy and relatively low cost. The CPE technique, compared to, e.g., the extraction in the Soxhlet apparatus (Table 1), is a very fast method, and the extraction time is between 10 and 20 min. Compared to other methods, the solvent consumption is also low, amounting to 5–10 cm^3^ per 1–50 g of raw material. In the context of its application to chemical analysis, the CPE technique can easily be combined with high-performance liquid chromatography (HPLC). The limitation of the method is that it cannot be used with a mass spectrometer detector, and the chromatographic column must be thoroughly cleaned of the surfactant. Moreover, the cloud points of some surfactants are very high, which precludes their use in the extraction of thermolabile compounds such as some vitamins [43].

The cloud point extraction, although was originally used only to determine inorganic compounds (metal ions), has found great application in the extraction of organic compounds (including impurities of organic origin) and biologically active substances, which are often ingredients in cosmetics or drugs. The latest application of the CPE technique also includes pro-environmental methods for the determination of nanoplastic [48] and nanometal [49] residues in the ecosystem, iron in beer [50], or the recycling of homogeneous catalysts from micellar solutions [51]. The CPE technique was successfully used to isolate active substances from plant and biological samples (blood, hair, urine, plasma, saliva) and also from food products. The CPE technique was used to extract biologically active substances such as flavones and flavanones [3,7,52], anthocyanins [52], triterpene saponins [42,53], vitamins A, E, K, B1 [18,43], paraffins [54], dyes [55,56], coumarins [57], anthraquinones [58], salicylic acid [18,43], paracetamol, flurbiprofen, or morphine [26,29,43], thymol [59], propyl gallate, butylated hydroxyanisole, and tert-butylhydroquinone [60], vanillin [39], lycopene, and other carotenoids [61]. Thus far, many non-ionic surfactants have been used, such as the very popular polyoxyethylene (7.5) octylphenyl ether (Triton X-100) or polyoxyethylene (9.5) octylphenyl ether (Triton X-114) [55,58,62], iso-tridecyl polyethylene glycol ether (Genapol X-080) [58], mixture of sucrose esters with fatty acids (SFAE surfactants), ethoxylated stearyl alcohol (Steareth-2 and Steareth-21), glycerin and polyethylene glycol stearate (PEG-5 glyceryl stearate), polyoxyethylene stearyl stearate (POE-5), fatty acid esters with glycerol and sorbitol, ethoxylated cetyl alcohol phosphate, glycerol stearate and polyethylene glycol stearate, a mixture of ethoxylated cetyl alcohol and stearyl alcohol (Ceteareth-6, stearyl alcohol, Ceteareth-25) [18], as well as silicone surfactants such as modified polydimethylsiloxanes (DC-190, DC-193) [56].

## 3. Nano-Micellar Extraction of Polyphenols

The following section provides an overview of the scientific publications, with several exceptions published between the years 2010 and 2020. Due to the application of the MME technique, the section is divided into two main subsections: analytical applications and practical use to obtain raw materials. Scientists who are deeply interested in the MME technique, especially in the context of other applications, are requested to refer to other very valuable reviews [5,18,29,40,43,46,52,63].

### 3.1. Analytical Application of MME—Advancement of Method and Examples of Use

The trace/ultra-trace concentration level of targeted compounds in real samples usually requires an initial step of isolation and/or preconcentration of analytes, especially in samples with complex matrix compositions. Conventional preconcentration approaches include liquid–liquid extraction (LLE) and solid phase extraction (SPE). However, these methods are associated with some disadvantages, such as being tedious and time-consuming, having a high consumption of organic solvents, and being expensive and labor intensive. Thus, to avoid LLE and SPE disadvantages, numerous new extraction methodologies are inclusive of solid-phase microextraction (SPME), stir-bar sorptive extraction (SBSE), single-drop microextraction (SDME), dispersive liquid–liquid microextraction (DLLME), hollow fiber–liquid-phase microextraction (HF-LPME), and cloud point extraction (CPE) have been developed [29,64,65].

The CPE is one of the most valuable preconcentration techniques used in analytical chemistry. Therefore, those surfactant-assisted techniques are often used to determine polyphenols in various samples. In such an application, the extraction of the analyte should be carried out under conditions that allow for the achieving of the highest value of the preconcentration factor (CF), and thus obtain the maximum extraction efficiency. This factor is defined as the ratio of the concentration of the extracted substance in the surfactant phase and its concentration in the initial aqueous surfactant solution (before concentration) [40,46]. To obtain the maximum value of CF, several factors should be taken into account: type, the concentration of surfactant, pH of the solution, ionic strength of the solution, extraction time, extraction temperature, centrifugation time. Over the last decade, many studies have been conducted on the determination of polyphenols with preliminary surfactant-mediated isolation/concentration of analytes; however, some with valuable achievements have been referenced below. In Table 2, the analytes, main characteristics of the extraction technique, and detection method are summarized.

In recent years, compounds of plant origin have gained a lot of interest, so it is not surprising that the compounds described in this section also mostly belong to this group. The largest group of analytes are flavonoids with the 2-phenyl chromone skeleton, and in particular, flavones, flavonols, isoflavones, and catechins. In addition, there are also phenolic acids. Two important naturally occurring types of phenolic acids are hydroxybenzoic acids and hydroxycinnamic acids, which are derived from non-phenolic molecules of benzoic and cinnamic acid. Another group of compounds are lignans, which are a large group of low molecular weight polyphenols found in plants, and particularly seeds, whole grains, and vegetables. Here, we also find phenol compounds originated from ginger, which activate spice receptors on the tongue and molecularly are a relative of capsaicin and piperine, compounds which are alkaloids. They are present in all members of the *Zingiberaceae* family. In Table 2 can be found also 1-deoxynojirimycin (DNJ), which is a polyhydroxylated piperidine alkaloid produced from D-glucose in various plants, and finally, synthetic phenolic antioxidants and preservatives.

As can be seen from the table below, the methods of detecting these analytes are classical: HPLC, UPLC-UV, HPLC-MS, MECK. The review indicates that the most important issue in the development of this type of analytical method seems to be the selection of an appropriate surfactant. The authors of discussed works have chosen among the classic compounds, i.e., cationic CTAB, anionic SDS, or the particularly popular non-ionic compounds such as Triton X-100, Triton X-114, or Genapol X-080. The use of ionic liquids (the IL-type surfactants), i.e., decylguanidinium chloride [66], C_16_C_4_Im-Br [67], C_12_mim-Cl [68], and biosurfactants, i.e., trehalose lipid [69], hyodeoxycholic acid sodium salt [70], or synthetic sugar surfactant APG0810 [71] are still the innovations in this field.

Khani et al. proposed also a very interesting improvement of the CPE [72]. This study was conducted for the quantitative determination of quercetin in food and fruit juice samples based on a green, fast, and accurate method, namely, micro-cloud point extraction (MCPE). The proposed MCPE is essentially a miniaturized form of traditional cloud point extraction (CPE) in which only a few microliters of the micellar extracting phase is sufficient for determination [72].

#### 3.1.1. Selection of the Appropriate Surfactant—Structure Effect

In all the works discussed in this section, more or less advanced optimization of the extraction methodology was carried out, using various theoretical approaches, such as single-factor assay, orthogonal experiment design, response surface methodology, and Doehlert experimental design. Effects of the surfactant concentrations, liquid–solid ratio, equilibration temperature, equilibration time, and salt addition on extraction yields were investigated and the optimal conditions have been established and summarized in Table 2. Below, we discuss in more detail the effects of the structure of the surfactants on the extraction efficiency.

Firstly, Mirzaei et al. proposed a simple and sensitive method for preconcentration and determination of genistein in soybeans based on cloud point extraction (CPE). To examine the surfactant type effect, a series of different non-ionic compounds, such as Triton X-100 (CMC = 189 ppm, 0.24 mM, HLB = 13.4 [80]), Triton X-114 (120 ppm, 0.168 mM, 12.4), Brij 700 (0.020 mM, HLB = 18.8), and Genapol X-080 (46 ppm, 0.05 mM, 13.0 [65]) were subjected to the same analytical procedure. The results demonstrated that maximum peak area was obtained for Genapol X-080 [77]. All tested surfactants are strongly hydrophilic and belong to the solubilizers. We observed also some correlation between the hydrophobicity of surfactants and the extraction efficiency, where the maximum analyte yield occurs for HLB = 13.0. It seems to be an optimal value but only for these analytes.

To find the optimal IL and evaluate its performance in the micellar extraction of six analytes of hawthorn fruit, 1-dodecyl-3-methylimidazolium-type ILs (C_12_mim) with different anions were tested by Hu et al. [68]. In this study, five long-chain ILs including [C_12_mim]Cl (CMC = 13.25 mM [80]), [C_12_mim]Br (9.26 nM [80]), [C_12_mim]CF_3_SO_3_, [C_12_mim]NO_3_, and [C_12_mim]HSO_3_ were compared as extraction solvents. Results indicated that the anions of ILs strongly affected the extraction yield. Moreover, they proved that the extraction efficiency of hydrophobic and hydrophilic compounds was largely anion-dependent. As shown, [C_12_mim]Cl exhibited the highest extraction yields for all investigated analytes [68]. The total content of polyphenols in the extracts decreased for the anions series: Cl^−^ > Br^−^ > NO_3_^−^ > CF_3_SO_3_^−^ > HSO_3_^−^, which clearly correlates with the Hofmeister (lyotropic) series of anions. Primary members of the series increase solvent surface tension and decrease the solubility of non-polar molecules (“salting-out”); in effect, they strengthen the hydrophobic interaction. By contrast, later salts in the series increase the solubility of non-polar molecules (“salting-in”) and decrease the order in water; in effect, they weaken the hydrophobic effect [81].

Zhou et al. developed a simple, inexpensive, and efficient method based on mixed cloud point extraction (MCPE) combined with high-performance liquid chromatography for the simultaneous separation and determination of six flavonoids (rutin, hyperoside, quercetin-3-O-sophoroside, isoquercitrin, astragalin, and quercetin) in leaf samples of *Apocynum venetum*. At the beginning of the study, Triton X-100 (CMC = 189 ppm, HLB = 13.4), Triton X-114 (120 ppm, 12.3), Triton X-45 (136 ppm, 9.8), and Genapol X-080 (46 ppm, 13.0) were evaluated as extraction solvents. The authors did not share these results, but indicated Genapol X-080, a relatively cheap and non-toxic surfactant, as best because of lack of absorption above 210 nm which caused no interference with the analyte signal [76]. This choice could be further rationalized because it is strongly hydrophilic (as is Triton X-100); however, it has the lowest CMC which allows minimally lower concentrations to be used. The authors further proposed to improve the method by using a mixture of surfactants. The addition of the cationic surfactant CTAB at a concentration 10 times lower than that of the non-ionic surfactant resulted in an almost 100% recovery of analytes. The authors attributed this effect to the formation of a neutral ion pair between negatively charged analytes and CTAB. Thus, the neutral ion pair can efficiently transfer to the surfactant-rich phase, compared to the absence of CTAB, leading to increased extraction recovery. Furthermore, the results show that with the MCPE approach, the peak shapes of the chromatogram are better (sharp, with good symmetry and no tail). At the same time, the peak height increased almost 3-fold, and therefore the enrichment factor increased almost 3-fold [76].

The structure of alkyl polyglucosides (APG) influences their physicochemical properties, which can affect the extraction efficiency of desired components [71]. The cited authors used two APGs differing in alkyl moiety, i.e., with hydrocarbon chain length 8–10 or 12–14 to obtain acceptable extraction yields of vitexin and vitexin-2’-O-rhamnoside from *Crataegus pinnatifida* leaves. The APG0810 (CMC = 823.8 mg/L) aqueous solution contained 60% alkyl polyglucosides, with a mean degree of polymerization (MDP) of 1.5. Meanwhile, the APG1214 (33.5 mg/L) was a 50% aqueous solution of alkyl polyglucosides with an MDP of 1.5. The 0.5% solution of APG0810 has comparable efficiency as ethanol; however, the 0.5% APG1214 was 4-fold less effective than alcohol. Of the two surfactants, the less hydrophobic one was proven to be better for the extraction of polyphenols. However, the authors did not try to use the third compound from the collection, i.e., APG0814 (CMC = 40.9 mg/L), and then probably some correlation would arise.

The effect of different biosurfactants on the extraction yield was evaluated by Peng et al. Authors used sodium chenodeoxycholate (CMC = 3.0 mM), sodium cholate hydrate (6.2 mM), sodium taurocholate (3–5 mM, 8–12 mM), sodium deoxycholate (2.4 mM), sodium *hyo*-deoxycholate (5 mM, 14 mM) and three chemical surfactants: SDS (8.2 mM), DTAB (14 mM), and Triton X-100 (0.22 mM). Compared to classic surfactants, the biosurfactant (*hyo*-deoxycholic acid sodium salt) showed higher extraction efficiencies for all the target analytes. The authors related this effect to the position of the hydroxyl groups in the sodium salt of *hyo*-deoxycholic acid. Some substitutions promote hydrogen bond formation and electrostatic interactions with analytes that further increase the mass transfer of the target analytes from sample powder to the aqueous phase [69]. Therefore, the hydroxyl group at the C6 position is quite unique when compared to the bile salts of human origin. The positional and stereochemical differences considerably influence micelle formation and solubilization ability [82]. Bile salts have smaller aggregation numbers of micelle compared with those of conventional aliphatic surfactants. In this case, the primary-secondary micelle model was considered, where the primary micelles are formed in such a way that the hydrocarbon backs of the steroid nucleus associate. The secondary micelles are then formed by the aggregation of these primary micelles. This model invokes a stepwise aggregation mechanism, i.e., polydispersity in the aggregates, where the critical micelle concentration (CMC) appears not as a point but over a certain concentration range. Cholates are not typical surfactants with the polar head and hydrophobic tail, but the whole molecule forms a kind of phase division plane, and the molecule includes hydrophilic and hydrophobic faces [83].

Du et al. extracted the main antioxidant compounds (geniposidic acid, chlorogenic acid, caffeic acid, and rutin) from functional plant tea (*Eucommia ulmoides* leaves). Several types of extraction solutions were chosen as potential extraction solutions for the BE-UAME procedure, including cyclodextrins (α-CD, β-CD, γ-CD, HP-β-CD, DIME-β-CD), glycolipids (sophorolipid (C16-18, sophorose), rhamnolipid (3-hydroxy fatty acid, rhamnose), trehalose lipid (particular acylated trehaloses with two fatty acids (sometimes iso) with 15 to 19 carbons, saturated or monounsaturated)), ethanol, 50% methanol, and ultrapure water. The results showed that the trehalose lipid solution was the most efficient extraction solution for each compound, and this was explained by the authors by the high number of hydroxyl groups in the trehalose lipid structure. In addition, the efficiencies for the other surfactants (α-CD, β-CD, γ-CD, HP-β-CD, and DIME-β-CD) or typical solvents such as ethanol, methanol, or ultrapure water were also not better than that of trehalose lipid. The research clearly shows that biosurfactants are better than methanol and ethanol, and slightly better than water [69]. In our opinion, the good performance of trehalose lipids was rather the result of its greatest hydrophobicity, but it is difficult to prove because of lack of data.

Finally, Moucková et al. tested a number of IL-based surfactants, i.e., [C_16_C_1_Im^+^][Br^−^] (logP = 3.19, HLB = 10.50, CMC = 0.61), [C_16_C_4_Im^+^][Br^−^] (4.51, 10.19, 0.10), [C_16_Py^+^][Br^−^] (3.47, 10.70, 0.72), [C_8_Gu^+^][Cl^−^] (2.09, 13.08 (10.18), 44.6), [C^10^Gu^+^][Cl^−^] (2.98, 12.13 (11.11), 18.6), and [(C_8_Im)_3_Bn_3_^+^]_3_ [Br^−^] (0.57, 8.69, 2.30) for the extraction of the three flavonoids rutin, quercetin, and apigenin from *Passiflora* sp. and *Mangifera* sp. leaves. One can see the proposed solubilizers are highly hydrophilic and moderately polar. Despite the authors’ efforts to find correlations, they were rather absent, except for the obvious conclusion that the surfactant should be selected for the particular plant material. Probably, a greater range of structural variation would allow a better assessment. The authors point out that [C_10_Gu^+^][Cl^−^] is a golden mean, that results rather from its low toxicity [66].

It is crucial to choose an appropriate type of extraction solution to enhance the efficiency of extraction of target analytes. Considering all the above data, it seems that the first choice, for those without experience, should be non-ionic surfactants with HLB in the range 11–13. However, in the context of analytical methods where the principle of the analyte is known, it seems that the hydrophilicity of the surfactant should be matched to the hydrophobicity of the compound(s) to be determined.

#### 3.1.2. Advantages and Disadvantages of the MME/CPE Approach in Analytics

The obvious advantages of CPE are as follows: (a) reduced extraction time; (b) low cost; (c) high-enrichment factor due to the small volume of surfactant-rich phase; and (d) the elimination of the use of toxic organic solvents. Interesting observations can be found in the work of Hu et al. [68]. The authors compare the efficiencies of micellar-based and conventional solvents (water and methanol) to extract six analytes from hawthorn fruit. It can be observed that water exhibited much higher extraction yields for the hydrophilic components than the hydrophobic ones, indicating itself to be an unsuitable extraction solvent for multiclass polar compounds. On other hand, the micellar solution and methanol had no significant difference in the extraction of target analytes from hawthorn fruit [68]. Moucková et al. achieved very promising results [66]. The work shows that it is possible to choose the conditions to extract selectively. The authors noted that rutin and quercetin require similar optimum conditions: low extraction times, temperature and l/s ratio, and the maximum IL-based surfactant concentration (50 times the CMC). In the case of apigenin, the best results were obtained with higher extraction times and l/s ratios, the highest extraction temperature, and the lowest IL-based surfactant concentration (CMC value) [66]. However, finally, considering its non-toxic and biodegradable characteristics, enhanced extraction capability, and environmental compatibility, biosurfactants are the most relevant as the extraction solution for the antioxidant components [69]. Moreover, in the context of the development of analytical methods, the application of the mixed-CPE approach could give positive improvements such as better chromatogram peak shapes (sharp, good symmetry, and no tailing) [75].

Considering the disadvantages, the environmental impact (biodegradation and toxicity) of ILs and ILBSs should be assessed. Thus, several studies on the relationship between their molecular structures and toxicity showed that the most toxic (to aquatic life) are those carrying aromatic/heterocyclic cations and long alkyl chains; most anions play a minor role in toxicity. Therefore, the synthesis of a new generation of easily biodegradable ILs and ILBSs from renewable sources was studied. It was shown that ester functionality enhances biodegradation of ILs; furthermore, adding a methyl group to the 2-position of the imidazolium cation and use of alkyl sulphate as a counter-ion also improves the biodegradability [80].

### 3.2. Practical and/or Technological Application of MME—Development of the Methodology and Examples of Use

An overview of the works from the last decade on the use of MME in the extraction of cosmetic, food, and pharmaceutical raw materials is summarized in Table 3. The research shows that for MME extraction, the most commonly used are ethoxylated fatty alcohols, e.g., series Triton^®^ from Union Carbide, Rokanol^®^ from PCC Group, or Brij^TM^ from Croda. The advantage of these surfactants is low surfactant concentration and high extraction efficiency. The following overview is organized to guide the reader from the most common application, such as extraction by solution of single non-ionic surfactants, to advanced mixed nano-micellar systems. We begin this description with two papers, where the authors studied the behavior of polyphenols in surfactant solutions using a range of physicochemical methods. We conclude this section with a summary of our team’s work.

Most of the published research on these compounds deals with nutritional, biochemical, or structural aspects. However, there are not many reports on the physicochemical properties, as well as the behavior of these substances in solutions. Löf et al. [84] proved that flavonoids such as naringenin, quercetin, and rutin can be solubilized in micelles and form very stable solutions. Research indicates that solubilization is related to the chemical structure of the studied flavonoids [84].

Another study aimed to investigate the interaction between the flavonoids quercetin and kaempferol and anionic twin surfactants (AOT and NaDEHP). Measurements of surface tension, absorption of UV and visible radiation, fluorescence, and measurements using the differential pulse voltammetry (DPV) method showed that in the case of AOT the phenyl substituent of the flavonoids is dissolved in the micelles. Whereas in NaDEHP micelles, the naphthyl residue was solubilized, which resulted in lower antioxidant activity of the tested flavonoids in AOT micelles. The research shows that the use of appropriate surfactants as a medium can regulate the antioxidant properties of the obtained extracts [85].

Chatzilazarou et al. [1] extracted polyphenols contained in the wine sediment using the cloud point extraction method with the use of non-ionic surfactants: Genapol X-080 and PEG 8000. The influence of the extraction process parameters on the process efficiency was investigated: time, surfactant concentration, pH, process temperature. The authors showed that the optimal time to achieve equilibrium is 30 min. The increase in polyphenol extraction efficiency was observed with the increase in surfactant concentration. Optimal values for obtaining a high content of polyphenols in the pseudophase are: 5% PEG 8000 and 2% Genapol X-080. The effect of pH on performance was also investigated in this system and it was stated that this parameter also had a significant effect on the results. Most polyphenols were extracted in the pH range from 2.5 to 3.5. A series of extractions at various temperatures ranging from 25 to 65 °C were also carried out. The optimum temperature turned out to be T = 55 °C. The authors of the study also showed, that in the case of polyphenols, it is important to use a temperature lower than 60 °C to avoid their degradation [1].

Stamatopoulos et al. [86] used the CPE technique to extract oleuropein and other polyphenols from olive leaves. A 4% aqueous solution of Tween 80 (polyoxyethylene (20) sorbitan monooleate) and the addition of Na_2_SO_4_ at 35% *w/v* were used. The process of extracting antioxidants was carried out for 5 min at the temperature of 25 °C, at the pH of the solution of 2.6. The analysis of the isolated compounds was performed by means of high-performance liquid column chromatography with the use of a diode detector. The recovery of the compounds was close to 100%. Moreover, the compounds isolated in the surfactant layer showed better thermal stability than the pure substances. After extraction, the antioxidant activity of the extracted polyphenols was still high. Thermal stability of polyphenols separated with pseudophase was investigated. It has been shown that it is greater compared to the polyphenols contained in the aqueous extract [86].

Katsoyannos et al. [6] used the cloud point extraction method to isolate polyphenols from aqueous olive oil extraction residue. The authors investigated the effectiveness of the method in the isolation of single polyphenols (tyrosol, syringic acid, gallic acid, protocatechic acid, coumaric acid, luteolin, oleuropein, rutin, apigenin) and their mixtures using Triton X-114. With a surfactant concentration of 4–6%, using a one- or multi-stage process, yields of over 96% were achieved for single polyphenols. Even 100% yield was obtained with luteolin. The authors showed that the amount of extracted polyphenols increases with increasing surfactant concentration. However, the accretion of said concentration was decreasing. Thus, for a higher concentration of polyphenols in the extracted sample, it is necessary to use a multistage process. By extracting the polyphenol mixture with a 6% surfactant solution in a one-step process, the researchers achieved a yield of less than 60%. Whereas in the three-stage process, a yield of over 90% was obtained using a 2% solution of Triton X-114 in each stage. It has also been observed that it is preferable to use lower surfactant concentrations. By extracting polyphenols from the post-process water using a 6% surfactant solution in a three-step process, the authors obtained a yield of just over 60% [6].

Tang et al. [87] used the CPE technique in conjunction with microwave-assisted extraction (MAE) to separate alkaloids and flavonoids from a Chinese plant called *Crotalaria sessiliflora* L. The highest extraction efficiency was observed with the use of 4% Triton X-100 solution and 1.4% sodium chloride addition. The most optimal temperature for heating the sample was 80 °C, with the extraction time up to 10 min and the solvent to plant material weight ratio 100: 1 (*w/w*). The resulting mixture was then centrifuged for one minute at a rotation speed of 3800 rpm. The aqueous phase was collected, and the surfactant phase was diluted with methanol to 5 cm^3^ and analyzed by HPLC. The extraction efficiency of vitexin, monocrotaline, and isovitexin was higher with the use of CPE than in the case of ultrasound-assisted extraction, where the process time was 60 min [87].

Jin et al. [88] received resveratrol from the peanut skin using microbial consortia immobilized on cellulose with an ultrasound-assisted 3% Triton X-114 aqueous system. The method of obtaining resveratrol was efficient, fast, green, and cheap for the extraction and bioconversion of target compounds from plant materials. The developed procedure could be a promising and effective method for producing resveratrol. The authors suggest this method could be widely used in producing targeting compound from plant waste residue in large-scale applications.

Miłek et al. [89] studied extracts from dandelion leaves and flowers, made with different solvent systems (aqueous acetone and Triton X-100 solution). Micellar-mediated extracts were analyzed for their antioxidant properties, polyphenol content, and the effect on live organisms. The research confirmed the significant antioxidant potential of extracts from the studied plants. The drawback of the method was the cytotoxicity of obtained extracts. However, cytotoxic effects may be desirable when a cancer cell line is being investigated.

To increase yield or reduce toxicity of extracting solution, the mixture of surfactant could be used [90]. Solvent-based extraction methods have a negative influence on mycorrhizal spore viability and vitality. The authors developed a biocompatible extraction method where spore and root viability are maintained with efficient extraction of rosmarinic acid. They screened temperature- and sonication-assisted techniques in ethanol, methanol, dimethyl sulfoxide, ionic liquid, and surfactants. Surfactants (Triton X-100 and Tween-20) at 1–3% were not found suitable for mycorrhizal viability.

Wu et al. [91] used folic acid-modified poly(ethylene glycol)-poly(ε-caprolactone) (Fa-PEG-PCL) nano-micelles, to encapsulate the luteolin. The authors create luteolin-loaded PEG-PCL (Lut/Fa-PEG-PCL) micelles to treat glioma both in vitro and in vivo. These Lut/Fa-PEG-PCL micelles induced a significant cell growth inhibition and more apoptosis of GL261 cells both in vitro and in vivo (compared with the free luteolin and Lut/MPEG-PCL).

Quercetin (QUE) is known to exhibit biological activity, including anti-cancer activity, but its low water solubility limits its clinical application. In order to improve the solubility and bioavailability of this bioflavonoid, Chen et al. developed a mixed polymer micelle (LMPM) system. The QUE-LMPM system was characterized by sustained release in in vitro studies [92].

Polyoxyethylene alcohol has also been used to obtain micellar extracts of *Bidens tripartita* L. (1% Rokanol NL5) for cosmetics application [93,94]. In micellar extracts, about twenty compounds from the group of polyphenols, mainly chlorogenic acid, caftaric acid and its derivatives, as well as luteolin 7-O-glucoside and luteolin, were identified by means of high-performance liquid chromatography (UPLC). On the other hand, when comparing the composition of the water extract of the trifoliate, obtained with the classical extraction method, it was found to lack 7-O-luteolin-glucoside and had a 3 times smaller amount of luteolin glucoside. The cosmetic preparations were assessed for their irritating properties on the EpiDerm^®^ cuticle model. The conducted research shows that the formulations are not irritating. The research shows that the micellar extract used in the composition has strong antioxidant properties. DPPH radical inhibition degree for the micellar extract was 73%. Additionally, the authors observed better antioxidant properties compared to ethanol (50%) and water extract (30%). The more favorable antioxidant properties of the micellar extract result from the higher content of the polyphenols that have anti-inflammatory, antiseptic, nourishing, and regenerating properties for atopic skin. In the next stage of the research, the analysis of the antioxidant properties of the finished cosmetic preparation, which is the subject of the invention, was performed. The emulsion (serum) showed strong antioxidant properties (62%), almost twice as high as the base emulsion without the extract (32%).

Recently, our team studied the connection between the chemical structure of non-ionic surfactants and the efficiency of the extraction process [11]. The effect of hydrophobic chain length and number of oxyethylene groups on the quality of three-part beggarticks extracts was investigated. Generally, aqueous surfactant solution utilization was unquestionably more efficient than water or ethanol extraction (polyphenols, flavonoids) from plant material. The highest total polyphenol content was determined in extracts with Oleth-10 and Oleth-5 and the highest total flavonoid concentration was obtained for Oleth-5. Efficiency of solubilization depends simultaneously on both structural modifications of surfactants: hydrophobic chain length and number of oxyethylene groups.

#### Theoretical Studies of Surfactant Aggregation and Polyphenol Solubilization

**Figure 5:**
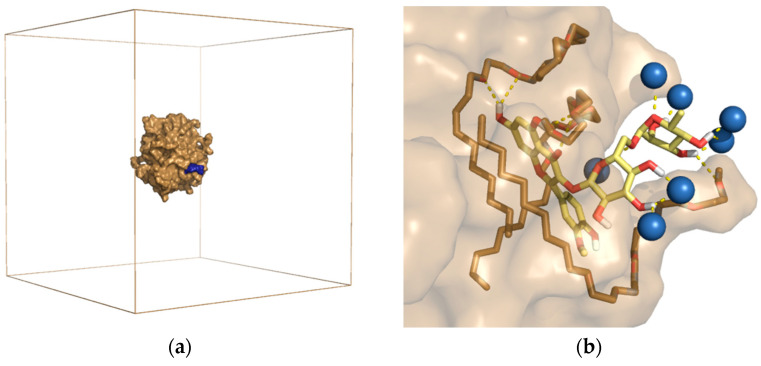
RNL5 micelle with adsorbed narcissin molecule after 100 ns of MD simulation. (**a**) shows the sand-colored micelle surface with blue-colored narcissin inside the simulation box. Water molecules are invisible. (**b**) Binding arrangement of narcissin (yellow) on the surface of RNL5 micelles. The water molecules (blue balls) and the surfactant (sand sticks) involved in the binding of the flavonoid are visible [10].

The results of experimental studies in recent years have confirmed that aqueous solutions of surfactants are effective in the extraction of polyphenols and flavonoids from plant material. It has been shown that non-ionic surfactants may have different dissolving power of active compounds depending on the structure and nature of the surfactant [3,7,9,10,96]. To analyze the phenomena occurring in the process of surfactant aggregation and the solubilization of flavonoids in a micellar solution, theoretical studies using the molecular dynamics method were also performed in our laboratory [9,10]. We used two models of surfactants: C9-11 Pareth-5 (RNL5) and PPG-4 Laureth-5. The results of the simulation of the spontaneous solubilization of one narcissin molecule in an aqueous solution of the surfactant (Figure 5) suggested that the process of narcissin solubilization is a multi-stage process and is determined by hydrophobic interactions. Moreover, it has been shown that an excessively large and/or branched polyether chain of the surfactant’s polar part can limit the diffusion of narcissin to the surface of the micelles. On this basis, it was concluded that probably not only the mass ratio of both parts of the surfactant (i.e., HLB) but also the structure of the hydrophilic part itself have an impact on the extraction efficiency [10].

In other research [9], we investigated the effect of hydrophobicity of polyphenols on their solubilization in an aqueous solution of surfactants. For this purpose, a series of simulations using the molecular dynamics method was carried out: solubilization of luteolin 7-O-glucoside and 3,5-O-dicaffeoylquinic acid (polyphenols in *Bidens tripartite* herb). The simulated surfactant was Rokanol^®^ NL5 (RNL5). The results of theoretical research proved that in the process of solubilization, polyphenols are incorporated into the Stern layer of micelles. The studied polyphenols are partially amphiphilic, and because they are structurally different, their binding method in micelles was slightly different. Although the main forces responsible for stabilizing the systems are predominantly hydrophobic, the role of electrostatic interactions cannot be ignored, especially in the case of flavonoid solubilization [9].

In the next publication [11], the effect of the number of oxyethylene groups in the structure of surfactants on the process of flavonoid solubilization was studied. Five models of ethoxylated decyl alcohol derivatives (Table 4), differing in a wide range of hydrophobicity, were selected for the study. Only C_10_H_21_(OC_2_H_4_)_5_OH (Rokanol^®^ NL5) is available on the chemical market, therefore no experiment was performed.

The simulation results showed that for all surfactants during the simulation, spontaneous adsorption of the flavonoid on the surface of the micelles occurred and then a single mixed micelle was formed. The exception was the most hydrophobic compound. To characterize the tested systems, the interaction energies between the luteolin glucoside and micelles were calculated, and the amounts of hydrogen bonds formed in these complexes, as well as the decrease in the degree of hydration of luteolin glucoside as a result of adsorption, were calculated. The results confirm that hydrophobic interactions are the main driving force for the solubilization of flavonoids. Based on experimental and theoretical research, it was proven that the surfactant must have a certain optimal ratio of the hydrocarbon chain length to the number of OE groups. For ethoxylated fatty alcohols used for the extraction of plant material, a ratio of 1.8 to 2.0 is recommended [11].

## 4. Conclusions

There is a great interest in the pharmaceutical, food, and cosmetic market in plant polyphenolic substances such as flavonoids due to their antioxidant activity. Therefore, it is not surprising to see an ever-growing number of scientific papers addressing both the extraction of these substances and the development of analytical methods. Effectively, we have been able to demonstrate in this review that the use of aqueous solutions of surfactants for the extraction/concentration of these compounds has a wide range of applications and has very many advantages. The above-mentioned studies mainly focused on obtaining specific substances or concentrating the analytes. However, there is still no systematic scientific research on the extraction of plant material by CPE. The general mechanism of solubilization is known, but the detailed analysis of the relationship between the concentration of the obtained active substance and the structure of the surfactant has not yet been fully elucidated. However, the latest research confirms the effectiveness of the CPE method in many applications. New perspectives on this topic are, of course, still working on the application of new types of surfactants, both classic and unusual ones. The development of computational and cheminformatic methods also allows us to look forward to further achievements in this aspect.

## Figures and Tables

**Table 1:** Comparison of CPE and other extraction methods (MAE—microwave-assisted extraction, UAE—ultrasound-assisted extraction, SFE—supercritical fluid extraction) [46].

Parameter	Extraction Method				
	CPE	Soxhlet Apparatus	MAE	UAE	SFE
Sample weight (g)	1–50	10–30	2–5	10–30	1–10
Type of solvent	aqueous surfactant solution	organic	organic	organic	CO_2_
Extraction time	10–20 min	6–24 h	20–30 min	30–60 min	30–60 min
Temperature	cloud point of surfactant	solvent boiling point	100–150 °C	30–35 °C	70–150 °C
Volume of solvent (cm^3^)	5–10	60–500	10–40	30–100	10–40
Pressure	atmospheric	atmospheric	atmospheric	atmospheric	15–50 MPa
Costs	low	low	medium	low	high

**Table 2:** Characteristics of surfactant-assisted extraction techniques used for determination of polyphenols.

Analyte	Method of Extraction/Sample	Extraction Conditions	Detection Method	Validation Parameters	Ref.
apigenin quercetin rutin	MAMME (IL-MA-SLE) passion fruit, mango leaves	decylguanidinium chloride, 525 μL, 930 nM; 50 mg; 30 °C, MV 10.5 min, 50 W	HPLC-PDA 340 nm; ACN/0.1% acetic acid, gradient, 1 mL/min; RP-18e (150 × 4.6 mm, 5 µm); 5 µL	LR = 0.05–500 mg/L, R^2^ > 0.9988; LOD = 10–40 µg/L	[66]
puerarin daidzein daidzin formononetin genistein genistin	CPE *Puerariae Lobatae* radix	Triton X-100, 0.07 g/mL; Liquid–solid ratio 80:1 (mL/g); NaCl 0.6 g; 70 °C, 40 min	HPLC 250 nm; MeOH/0.2% phosphoric acid, gradient, 1 mL/min; C18 (4.6 × 250 mm, 5 µm); 35 °C, 20 µL	LR = 0.8–1000 µg/mL, R^2^ > 0.99; LOD = 15.2–30.7 ng/mL, LOQ = 50.6–102.4 ng/mL; Recovery: 95.12–103.65%	[73]
caffeic acid chlorogenic acid geniposidic acid rutin	BE-UAMME *Eucommia ulmoides* leaves	trehalose lipid solution, 10 mL,3 mg/mL; 100 mg; US 35 min	UPLC-DAD 237–360 nm; ACN/0.1% formic acid, gradient, 0.3 mL/min; C18 (2.1 × 100 mm, 1.7 μm); 50◦C, 1 μL	LR = 0.80–200 μg/mL, R^2^ > 0.9999; LOD = 0.6–1.0 µg/g	[69]
caftaric acid quercetin quercetin-3-O-glucoside querceting-3-O-glucuronide rutin	IL-MA-SLE *Vitis vinifera* leaves	C_16_C_4_Im-Br, 2.25 mL, 0.1 mM; 0.100 g; 70 °C, MV 30 min, 50 W; 2504× *g*, 5 min	HPLC-PAD 360 nm; ACN/0.1% *v/v* acetic acid, gradient, 1 mL/min; RP-Amide (15 cm × 4.6 mm × 5 µm); 5 µL	LR = 5–500 mg/L, R^2^ > 0.992; LOD = 0.5–3.0 mg/L, LOQ = 1.7–5.0 mg/L	[67]
quercetin	MCPE onion, tomato, apple, orange juice	Triton X-114, 1 mL, 5% *v*/*v*; Na_2_SO_4_, 1.3 mL, 5% *w*/*v*; 40 °C, 10 min; 5000 rpm, 6 min	UV-Vis	LR = 10–100 ng/mL, R^2^ = 0.9994; LOD = 2.2 ng/mL	[72]
astragalin chlorogenic acid deoxynojirimycin isoquercitrin rutin	UASE-CPE mulberry leaves	Triton X-114, 3%; Liquid–solid ratio of 35:1; 0.05 M HCl; US 45 min, 360 W	HPLC 360 nm; ACN/0.1% formic acid, gradient, 1 mL/min; PFP 5u (250 × 4.6 mm, 5 µm); 30 °C, 20 μL	LR satisfactory, R^2^ ≥ 0.99; LOD = 0.58 mg/mL, LOQ = 1.87 mg/mL	[74]
arctigenin caffeic acid forsythoside phillyrin isorhamnetin quercetin	ND-VSMSPD *Forsythiae Fructus*	Triton X-114, 2 mL 10% (*v/v*); Florisil, sample/sorbent ratio 1:1; grinding, 3 min; whirling, 2 min	UHPLC 280 nm; ACN/0.1% formic acid, gradient, 0.3 mL/min; C18 (2.1 × 100 mm, 1.7 μm); 30 °C, 1 μL	LR = 0.08–50 μg/mL, R^2^ ≥ 0.999; LOD = 0.03–0.08 μg/mL, LOQ = 0.08–0.25 μg/mL; Recovery: 95–104%	[75]
6-gingerol 8-gingerol 10-gingerol 6-shogaol zingerone	UAME and MAME *Rhizoma Zingiberis* and *Rhizoma Zingiberis Preparata*	hyodeoxycholic acid sodium salt, 100 nM; 60 °C, MV 10 s	UHPLC 280 nm; ACN/0.1% formic acid, gradient, 0.4 mL/min; SB-C18 (1.8 μm, 50 × 4.6 mm); 35 °C, 2 μL	LR = 1–100 μg/mL, R^2^ = 0.9995–0.9998; LOD = 3.80–8.11 ng/mL, LOQ = 12.5–26.8 ng/mL; Recovery: 87.32–103.12%	[70]
quercetin	HF-LLME-RM plasma, onion, tomato	CTAB, 7 mmol/L; pH = 7.5, 1-octanol; rt, 900 rpm, 30 min	HPLC 370 nm; methanol/0.3% phosphoric acid, 58:42, 1.0 mL/min; C18 (5μm, 150 × 4.6 mm); 20 μL	LR = 0.5–1000 ng/mL, R^2^ = 0.9992; LOD = 0.1 ng/mL, LOQ = 0.33 ng/mL	[64]
vitexin vitexin-2′-O-rhamnoside	UAE *Crataegus pinnatifida* leaves	APG0810,10 mL, 0.7%; Liquid–solid ratio 25 mL:g, soaking 2 h, US 34 min, 250 W, 50 kHz	HPLC 360 nm; THF/ACN/MeOH/0.5% acetic acid (17.1:1.7:1.2:80, *v/v*/*v/v*), 1 mL/min; C18 (5μm, 150 × 4.6 mm); 25 °C, 10 μL	LR = 0.03–0.50 mg/mL, R^2^ = 0.9992–0.9997; Recovery: 96.1%, 103.4%	[71]
astragalin hyperoside isoquercitrin quercetin quercetin-3-O-sophoroside rutin	MCPE *Apocynum venetum* leaf	Genapol X-080, 1.2% *w/v*, CTAB 0.1% *w/v*; pH = 8, sodium chloride 1.0% (*w/v*); 0.1 g; 55 °C, 10 min; 4000 rpm, 5 min	HPLC-DAD 360 nm; ACN/0.1% phosphoric acid– 0.05% triethylamine, gradient; SB-C18 (3.5μm, 100 × 4.6 mm); 25 °C, 10 μL	LR = 20.0–1000.0 ng/mL, R^2^ > 0.9994; LOD < 5.0 ng/mL, LOQ < 20.0 ng/mL, Recovery: 93.9%–98.8%	[76]
chlorogenic acid epicatechin hyperoside isoquercitrin protocatechuic acid quercetin	IL-UAMME *Crataegus pinnatifida* fruits	[C_12_mim]Cl, 20 mL,150 mM; 1 g; US 40 min, 100 W	UHPLC-Q-TOF/MS ACN/0.1% formic acid, gradient, 0.4 mL/min; Ext-C18 (2.1 × 50 mm, 1.8 μm); 35 °C, 1 μL	LR = 0.1–40 μg/mL, R^2^ = 0.9934–0.9999; LOD = 3.0–5.4 ng/mL	[68]
genistein	UACPE soybeans	Genapol X-080, 25 mL, 5% *v/v*; 1 g; 40 °C, US 45 min	HPLC 254 nm; 5% ACN/ 0.1% acetic acid (*v/v*) in ACN, gradient, 1 mL/min; SB-C18 (250 × 4.6 mm, 5 µm); 25 °C, 20 µL	LR = 0.1–10.0 µg/mL, R^2^ = 0.9964; LOD = 15.0 ng/mL; RSD(7) = 4.45%	[77]
quercetin quercitrin rutin	SA-PLE *Costus speciosus*	SDS 0.2% *w/w* or Triton X-100 0.1% *v/v*; 0.5 g; 1.5 mL/min, 30 min, 20–30 bar	MEKC capillary, 51.5 cm/60 cm, 76 μm; 10 mM phosphate, 10 mM borate, 50 mM SDS, pH 8.5; 20 kV; 370 nm; 20 °C, 50 mbar, 5 s	LR = 10-100 mg/L, R^2^ > 0.994; LOD = 0.32–0.69 mg/L, LOQ = 1.07–2.30 mg/L,	[78]
butylhydroxyanisole butylhydroxytoluene propyl gallate tert-butylhydroquinone	CPE edible oil	Triton X-114, 2.5% *v/v*; NaCl 0.5% *w/v* 50 °C, 40 min	HPLC 280 nm; MeOH/1.5% acetic acid, gradient, 1 mL/min; TC-C18 (150 × 4.6 mm); 40 °C, 20 μL	LR = 1–500 μg/mL; LOD = 1.9–11 ng/L, Recovery: 81–88%; CF = 14	[60]
ampelopsin	CPE rat plasma	Genapol X-080, 1 mL, 5%*w/v*; NaCl, 100 mL, 0.6 M; 55 °C, 20 min; 3500 rpm, 10 min; acetonitrile–water, 200 μL, 30:70, *v/v*; 16 000 rpm, 5 min	HPLC 290 nm; ACN/0.1% phosphoric, gradient, 1 mL/min; SB-C18 (150 × 4.6 mm, 5 μm); 25 °C, 20 μL	LR = 20–2000 ng/mL, R^2^ = 0.9996; LOD = 6 ng/mL, LOQ = 20 ng/mL	[79]

**Table 3:** Characteristics of surfactant-assisted extraction techniques used to source polyphenols.

Polyphenol	Extraction Conditions	Advantages	Disadvantages	Ref.
luteolin	Triton X-100 2% *v/v* t = 30 min	good performance compared with 30% acetone	cytotoxicity of extract	[89]
vitexin, isovitexin, and monocrotaline	Triton X-100 5% *v/v* T = 80 °C, t = 60 min	higher extraction efficiency with the use of CPE	long time of the ultrasound-assisted extraction (60 min)	[87]
glycyrrhizic acid, liquiritin	Triton X-100 5% *v/v* T = 99 °C, t = 3–5 min	the extraction efficiency approached 100%, effective, rapid method-coupling of microwave-assisted extraction and cloud point extraction	-	[95]
rosmarinic acid	Triton X-100, Tween-20 3% *v/v* T = 30 °C, t = 15 min	-	surfactants less efficient eluent than 10% methanol, 0.25 M ionic liquid and dimethylsulfoxide	[90]
tyrosol, syringic acid, gallic acid, protocatechic acid, coumaric acid, luteolin, oleuropein, rutin, apigenin	Triton X-114 6% *v/v*	high yield 96%	for a higher concentration of polyphenols in the extracted sample- multistage process	[6]
resveratrol	Triton X-114 3% T = 30 °C, t = 36 h, 250 W liquid/solid = 25:1 mL/g	high performance, 4-fold to that of untreated sample, enhancer to stimulate the cell to produce more enzyme	-	[88]
naringenin, rutin, quercetin	Tween 80	naringenin and rutin solubilized in the Tween 80	quercetin not solubilized in the Tween 80	[84]
polyphenols	Tween 80 4% *v/v* T = 25 °C, t = 5 min pH = 2.6	thermal stability of polyphenols	-	[1]
antioxidants	Tween 20, 40, 60, 80 1% *w/v* T = 25 °C, t = 30 min	-	Tween 20 not sufficient to obtain antioxidants	[96]
thymol	Span 80, 30% *v/v* T = 65 °C, t = 45 min	-	-	[59]
kaempferol, quercetin	Sodium bis (2- ethylhexyl) sulfosuccinate, sodium bis (2-ethylhexyl) phosphate	radical scavenging activity and degradation rate constant of flavonoids higher in NaDEHP micelles as compared to AOT micelles	-	[85]
quercetin	Lecithin, Pluronic^®^ P123, and 1,2-distearoyl-sn-glycero-3-phosphoethanolamine-N-methoxy [poly (ethylene glycol)-2000	the solubility of quercetin in the LMPM system higher compared to that in water	-	[92]
lycopene	Genapol X-080 5% *v/v* T = 55 °C, t = 30 min pH = 2.3	high yield 92.3%	for a higher concentration of polyphenols in the extracted sample-multistage process	[61]
polyphenols, flavonoids	Rokanol B2, Triton X-100 Tego Care CG 90, Crodesta F160, WPC 1%*w/v* T = 25 °C, t = 30 min	higher contents of total flavonoids and polyphenols in the micellar extracts. The whey proteins could be the effective agents for MME	-	[3]
isookanin 7-O-glucoside, luteolin 7-O-glucoside, luteolin, chlorogenic acid, caftaric acid	Rokanol B2, Triton X-100, Tego Care CG 90, Crodesta F160, WPC 1%*w/v* T = 25 °C, t = 30 min	the selection of a suitable surfactant may thus pro- vide the expected composition of extract	-	[7]
chlorogenic acid, caftaric acid, luteolin 7-O-glucoside, and luteolin	Rokanol NL5 1%*w/v* T = 25 °C, t = 30 min	better antioxidant properties compared to ethanol and water extract, low irritating potential of the micellar extract, very good antioxidant properties of the cosmetic with micellar extract compared to the formulation with water extract	-	[10]
chlorogenic acid, di-O-caffeoylquinic acid	Rokanol NL5, B2, L4P5 1%*w/v* T = 25 °C t = 30 min	the biggest micelles obtained in the case of NL5, the best solubilization agent	-	[9]
chlorogenic acid luteolin 7-O-glucoside	BrijTMCS20, BrijTMS20, BrijTMO20, BrijTMO10, BrijTMO05 1%*w/v* T = 25 °C t = 30 min	initially nano-micellar systems, very low surfactant concentration and high extraction efficiency, not irritating extract	-	[11]

**Table 4:** Structure of tested surfactant models. Calculated HLB indices.

Surfactant Model	HLB Griffin
C_10_H_21_OC_2_H_4_OH	6.04
C_10_H_21_(OC_2_H_4_)_3_OH	10.27
C_10_H_21_(OC_2_H_4_)_5_OH	12.54
C_10_H_21_(OC_2_H_4_)_7_OH	13.95
C_10_H_21_(OC_2_H_4_)_10_OH	15.28
C_10_H_21_(OC_2_H_4_)_15_OH	16.55
